# Oligometastatic Prostate Cancer—The Middle Child Syndrome

**DOI:** 10.3390/jcm12237198

**Published:** 2023-11-21

**Authors:** Ee Jean Lim, Mengyue Su, B. M. Saiduzzaman, Kae Jack Tay, Henry Sun Sien Ho, Theodoros Tokas, Bhaskar Kumar Somani, Vineet Gauhar, John Shyi Peng Yuen, Kenneth Chen

**Affiliations:** 1 Department of Urology, Singapore General Hospital, Singapore 169608, Singaporekenneth.chen@singhealth.com.sg (K.C.); 2 Department of Urology, University General Hospital of Heraklion, University of Crete, Medical School, 14122 Heraklion, Greece; 3 Training and Research in Urological Surgery and Technology (T.R.U.S.T)—Group, 6060 Hall in Tirol, Austria; 4 Department of Urology, University Hospital Southampton NHS Foundation Trust, Southampton SO16 6YD, UK; 5 Department of Urology, Ng Teng Fong Hospital, Singapore 609606, Singapore

**Keywords:** oligometastatic prostate cancer (OMPC), metastasis-directed therapy, imaging

## Abstract

Oligometastatic prostate cancer is an evolving clinical entity as more data from novel imaging tools such as PSMA PET/CT emerges. Recognition of this disease entity allows for unique interventions which differ from conventional treatment of metastatic prostate cancers such as the initiation of chemotherapy. With metastasis-directed therapy (MDT), there is potential for early eradication of limited disease metastases and a delay in systemic treatment with its associated treatment-related toxicities. This review explores the current evidence and outcomes of different metastasis-directed therapies such as the role of radiotherapy in low volume metastasis and the use of PSMA ligands to facilitate pelvic lymph node dissections. With a deeper understanding of this low metastasis state, it has revolutionized the current viable treatment options, and more studies are ongoing to provide further insights into this unique disease entity.

## 1. Introduction

Prostate cancer is a significant global health concern, with varying prevalence rates across different regions. It is the most common cancer among men worldwide, excluding non-melanoma skin cancers [1]. With a significant burden of disease, prostate cancer management has steadily matured throughout the years with evolved treatment pathways for the primary localized disease, locally advanced disease, oligometastatic cancer, and high-volume metastatic disease. There is a wide spectrum in tumour biology, from indolent cancers to aggressive phenotypes with high metastatic potential. Prostate cancer treatment is primarily dictated by the stage and International Society of Urological Pathology (ISUP) grade group [2,3].

Risk stratification systems have been developed by various cancer and urological organizations such as the National Comprehensive Cancer Network (NCCN, USA), National Institute for Health and Clinical Excellence (NICE, UK), European Society of Medical Oncology (ESMO), American Urological Association (AUA), and the European Association of Urology (EAU), which are risk stratifying patients by grade group and their serum prostate-specific antigen levels (PSA) into low-risk to very high-risk, with staging imaging indicated for patients with an unfavourable-intermediate risk and above [1,4,5].

The concept of “oligometastatic disease” was first described in 1995 by Hellman and Weichselbaum, to identify a subgroup of metastatic patients with a limited number of clinically detectable metastases. It may represent a more indolent disease entity in which focal metastasis-directed therapies (MDTs) can be utilized to prevent further disease spread, delay systemic therapy, and potentially improve overall survival. The theory behind MDTs in this setting is to aim to eliminate the oligometastatic colony before it can evolve biologically into a more aggressive phenotype with a poorer prognosis, including potential tumour seeding of other sites [6].

## 2. Definition of Oligometastatic Prostate Cancer

Oligometastatic prostate cancer (OMPC) is an intermediate biological state with a unique clinical picture nested within a spectrum of advanced disease, which is continually redefined as novel imaging tools are introduced and adopted. But before discussing the impact of novel imaging tools, a review of the current definition of OMPC is in order.

There is no consensus on the definition of oligometastatic prostate cancer with respect to the number or location of metastases [7]. For patients with a prostate cancer diagnosis, the two most commonly utilized and widely referenced definitions of disease volume are extrapolated from the LATITUDE and CHAARTED clinical trials. The LATITUDE trial defined high risk as having two or more of the following criteria: ≥3 bone metastases, visceral metastases, and ≥ISUP grade 4. The CHAARTED trial defined high-volume disease as ≥4 bone metastases (including ≥1 beyond the vertebral column or pelvis) or visceral metastases. However, there are slight nuances when considering the entities of the low-volume metastatic disease and oligometastatic disease.

The concept of oligometastatic disease was borne with the ideation of MDTs while disease volume was considered when attempting to identify a subset of the population with metastatic disease who would fare better prognostically while on systemic treatment. Therefore, the former definition is a pragmatic one looking at disease characteristics that could guide local therapy, while the disease volume may focus more on parameters weighted towards prognosis of patients on systemic therapy. Therein lies the differences in how location and number are interpreted. The definition of oligometastatic disease, as outlined in both the Advanced Prostate Cancer Consensus Conference and the Italian Association of Radiotherapy and Clinical Oncology and Radiotherapy consensus documents, is centred on the presence of bone and lymph node involvement, with a maximum of three synchronous metastases in these areas, while excluding any mention of visceral lesions [8,9]. Gandaglia et al. found that the median overall survival was 43 months for LN metastases, 24 months for bone metastases, 16 months for visceral metastases, and 14 months for bone plus visceral metastases, where visceral metastases carry significantly higher risks of overall cancer-specific mortality versus those with exclusively LN metastases. The size and location of the metastasis can reflect the underlying disease biology and can be pivotal in determining the appropriate metastasis-directed therapy (MDT) technique [10].

There is convincing evidence demonstrating improved survival in patients with less than five lesions compared to patients with five or more lesions [11]. Hence, with this backdrop, the ideal oligometastatic patient who would have optimal outcomes with MDT, with or without systemic treatment, is probably one with three non-visceral metastatic lesions, while understanding the potential expansion of this numerical criterion to four. The exclusion of visceral metastases is more certain regardless of whether we are considering the feasibility of local control or whether we are prognosticating the metastatic patient.

Indeed, as we gain more understanding of metastatic prostate cancer and the outcomes in relation to disease burden, the concept of high and low volume has taken on a new meaning. The criteria used in the CHAARTED trial rely on factors such as the presence of the visceral disease in CT scans and the number and location of bone metastases in bone scans. Inconceivably, using this definition, patients with only lymph node metastases are classified as having a low burden, regardless of the extent of the nodal disease. The initial analysis of the STAMPEDE trial indicated that the survival benefits of prostate radiotherapy gradually decreased as the number of visible bone metastases in the baseline bone scans increased [12]. This may suggest that a simple enumeration of metastases would be a simpler method to use when optimizing the selection of patients for local therapy. And to confound matters, next generation imaging now seeks to identify the micrometastatic disease at an earlier stage, which really begs for a rethink of our definition of disease burden.

## 3. Imaging

Up until now, trials have utilized conventional imaging modalities such as CT scans, MRI scans, and 99mTc-methyl diphosphonate (MDP) skeletal scintigraphy. The introduction of next molecular imaging in recent years have engendered a new clinical conundrum even before we have resolved an earlier one, with the undisputed superiority of PSMA PET in staging prostate cancer [13].

With next generation molecular imaging and reclassification of disease stages, we are seeing the emergence of a Will Rogers’ Phenomenon, which describes the statistical effect that occurs when redefining the criteria for a disease, typically by using more sensitive diagnostic methods, causing patients to be reclassified from one disease category to another. This can lead to an apparent improvement in the outcomes of both groups, even though the overall disease prevalence remains unchanged. Indeed, some high-risk localized prostate cancer patients, initially negative for metastasis on conventional imaging, are now being reclassified as part of the metastatic patient cohort when PSMA PET/CT detects oligometastases. This stage migration invariably improves outcomes for the localized disease group by removing those who actually have oligometastatic conditions, and concurrently, it enhances the survival for the cohort of metastatic patients by incorporating individuals with a low-volume metastatic burden [14].

However, despite the decisional change that this situation brings about, it remains uncertain whether the management impact would lead to better oncological outcomes. But the fact remains that a subgroup of patients with limited low-volume metastatic burden exists and may present an opportunity for us to extend curative care and de-escalate systemic treatment.

## 4. Clinical Impact

The importance of defining the entity of OMPC lies in the opportunities for intervention at this stage of the disease spectrum. Indeed, the management of OMPC is unique and deviates from conventional treatment of newly diagnosed advanced prostate cancer in a few ways [15].

### 4.1. Metastasis Burden Guides the Use of Chemotherapy

Firstly, the metastatic burden does influence the choice of treatment. Treatment intensification has become the new standard of care for metastatic hormone sensitive prostate cancer (mHSPC) [12,16,17].

Several landmark trials investigating the combination of ADT with androgen pathway receptor inhibitors (ARPIs) have established level 1 evidence for the survival benefit not just in the setting of high-volume but also low-volume metastatic burden. In contrast, the use of chemotherapy has not been shown to always be beneficial in the setting of low-volume metastases [2,14,16,18]. Although there is a consensus regarding the benefits of docetaxel in high-volume metastatic disease, GETUG-AFU 15, CHAARTED, and STAMPEDE presented contrasting results regarding the use of chemohormonal combination therapy in patients with low metastatic burden [19,20,21].

The divergent outcomes among these trials can be partly attributed to differences in the study population and treatment protocols. For instance, both CHAARTED and GETUG-AFU 15 had fewer de novo mHSPC patients compared to the STAMPEDE trial. Nonetheless, the findings of at least two meta-analyses have concluded that the overall survival benefit that combination ADT and docetaxel offers is limited to the high-volume disease [22,23].

This underpins the importance of defining the metastatic burden, with consideration to genomics/biomarkers, patient status, and preferences, which can guide the appropriate use or in this case, avoid the inappropriate use of chemotherapy in OMPC patients.

### 4.2. Metastasis-Directed Therapy (MDT)

Notwithstanding the survival benefits of combination treatment of ADT with ARPIs, aggressive castration has long-term adverse effects like bone density loss and cardiovascular risks. Metastatic directed therapy (MDT) offers the potential to reduce these toxicities by delaying the progression of metastatic disease and in some situations, eradicating them. Exerting targeted control over these sites of early disease spread also allows the delay of systemic treatment which in turn limits a patient’s duration of androgen suppression and treatment related toxicities [3,19].

There are several approaches to management of oligometastases, and the modalities available include metastatectomy, stereotactic body radiation therapy (SBRT), or a combination of these therapies with or without ADT. However, current studies suffer from small numbers, heterogenous inclusion criteria, and a lack of standardized outcome measures, as well as long-term data. Hence, a universal approach and consensus is still lacking [3,24].

Among the different strategies, SBRT has the largest body of evidence and several systematic reviews and meta-analyses (SRMA) offer insights into its effect in OMPC. Ost et al. showed that for metachronous OMPC patients who underwent salvage MDT (RT 66%, lymph node dissection 34%), a 3-year PFS of 51% could be achieved, although the majority (61%) had adjuvant ADT [3]. There were more grade 2 complications from LND compared to RT (11% vs. 8.5%). In another SRMA that included 653 patients from 10 studies with metachronous OMPC (3–5 lesions based on new-generation imaging with PET/CT) treated with SBRT, the 2-year biochemical PFS, rPFS, and ADT-free survival were 33% (95% CI, 11–55%), 39% (95% CI, 24–54%), and 52% (95% CI, 41–62%), respectively. Another SRMA assessing the efficacy of SBRT for oligometastatic PCa recurrence reviewed data from 23 studies and 1441 treated lesions, showed PFS and ADT-free survival of 0.413 (95% CI, 0.378–0.477) and 20.1 mo (95% CI, 14.5–25.6), respectively. The authors also demonstrated a dose-dependent relationship with rates of local control, along with low rates of toxicities [7,12]. A pooled analysis of the only two prospective randomized trials in MDT (STOMP and ORIOLE) provided insights into the long-term outcomes of MDT and showed a sustained benefit in the median PFS with MDT compared with observations (pooled hazard ratio [HR], 0.44; 95% CI, 0.29 to 0.66; *p* value < 0.001). High-risk mutations (somatic mutations within ATM, BRCA1/2, Rb1, or TP53) further risk stratified these patients with an improved PFS in those without the mutations (PFS 13.4 months vs. 7.5 months; HR 0.53; 95% CI, 0.25 to 1.11; *p* = 0.09) [25]. Whether conventional imaging or newer molecular imaging such as PSMA is used, the data show a trend towards the clinical benefits of MDT, and ongoing trials will further consolidate the experience with MDT in OMPC (Table 1).

### 4.3. Radiotherapy to Primary Tumour in Low Volume Metastasis

The approach to treatment intensification has traditionally centred around combining various pharmacological agents. However, it is essential to recognize that this strategy can also encompass a broader spectrum of multimodal treatment, involving both systemic therapies and localized treatments aimed at the primary tumour [2,26].

In the context of initiating treatment for metastatic hormone-sensitive prostate cancer (mHSPC), the pivotal HORRAD trial delved into the potential benefits of prostate radiotherapy. This trial enrolled 432 patients who were randomized into either receiving ADT alone or ADT in combination with intensity-modulated RT targeted at the prostate. Notably, while there was no observed overall survival advantage (HR: 0.9 [0.7–1.14]), the arm receiving radiotherapy showed a significant improvement in the median time to PSA progression (HR: 0.78 [0.63–0.97]) [17].

Parallel findings emerged from the STAMPEDE trial, wherein survival benefits were not universally apparent among the unselected patient population. However, an evident advantage in both the overall and biochemical recurrence-free survival was distinctly shown within the subgroup of low-volume metastases (as per CHAARTED criteria). In the most recent final analysis, with a median follow up of 61.3 months (interquartile range [IQR] = 53.8 to 73.1), which was similar in both treatment groups, the improvement of OS in men with newly diagnosed, low-burden metastatic prostate cancer but not in men with high-burden disease was confirmed. In the low metastatic burden group, the point estimate for HR of OS has improved slightly from 0.68 to 0.64 (95% CI 0.52 to 0.79; *p* < 0.001 [*p* = 0.00004]) for men in the low metastatic disease risk group compared to the initial analysis. The differential treatment effect according to metastatic burden was distinct and significant: interaction test *p* < 0.001 [*p* = 0.00005]. This survival benefit comes without the negative impact to the QoL. There was no significant difference in time to local symptoms, nor was there any evidence of a difference in the Global QoL or QLQ-30 Summary Score [33].

This important observation was further substantiated by a comprehensive meta-analysis spanning both trials. Consequently, this collective body of evidence has firmly established a gold standard of care in the local control of the primary tumour with radiotherapy for patients characterized by synchronous low-volume metastases [34].

### 4.4. Cytoreductive Prostatectomy

The benefits of local therapy to the primary tumour in the metastatic setting is not limited to stereotactic radiotherapy. There is a growing body of evidence surrounding cytoreductive prostatectomy. Evidence supports the potential therapeutic benefits for cytoreductive surgery in certain cancer types such as metastatic renal cell carcinoma and ovarian cancer. For breast cancer, cytoreductive surgery has resulted in an improved overall survival in patients with metastatic disease [35]. These efforts to define the utility of cytoreduction surgery in cancer treatment are based largely on the “seed and soil” theory which proposes that the primary tumour can act as a constant source of circulating tumour cells with the potential to propagate tumour growth both locally and distantly [35,36].

With prostate cancer, despite mouse studies demonstrating the benefits of improved PSA-progression rates, metastasis-free rate, and prolonged survival following cytoreductive surgery [37,38], cytoreductive surgery remains a topic of controversy. This is likely due to the lack of randomized controlled trials investigating its efficacy in the setting of metastatic prostate cancer. Nonetheless, survival benefits of cytoreductive prostatectomy (cRP) have been confirmed by at least two meta-analyses [39,40]. In the most recent one, which included 10 studies looking at a total of 888 patients, cytoreduction of the primary prostate tumour was found to significantly improve in both the long and short term [OR = 1.77, 95% CI (1.01, 310), *p* = 0.04] and [OR = 2.71, 95% CI (1.72, 4.29), *p* < 0.0001], as well as progression-free survival [OR = 1.93, 95% CI (1.25, 2.97), *p* = 0.003]. Nonetheless, the results of this pooled analysis have to be moderated in light of limitations such as a lack of high-quality evidence and heterogenous inclusions of included studies. Other prospective studies have added valuable evidence to the body of knowledge. Dai et al. reported on the prospective phase 2 RCT FUSCC-OMPCa trial, investigating the survival benefits of radical local therapy (RLT) beyond androgen deprivation therapy in newly diagnosed oligo-mHSPC patients [41]. The majority of patients randomized to radical local therapy had a cytoreductive prostatectomy (85%). Over a median follow-up of 4 years, the authors demonstrated that when RLT was added to ADT, the risk of radiographic progression was reduced by 57% (HR 0.43, 95% CI 0.27–0.70, *p* = 0.001). Moreover, the addition of RLT to ADT was also associated with a 56% reduction in the risk of death [HR of 0.44; 95% CI of 0.24–0.81; *p*-value = 0.008]. Data from the prospective multicentre Belgian Local Treatment of Metastatic Prostate Cancer (LoMP) registry, which includes patients with low-volume mHSPC treated with local therapy (cytoreductive radical prostatectomy or radiation therapy) also offered interesting insights. Over a 32-month median period, a group of patients who received cytoreductive radical prostatectomy in addition to ADT showed better 2-year overall survival and cancer-specific survival rates compared to those who received systemic therapy alone (93% vs. 69% and 93% vs. 75%, respectively). Nonetheless, these results are limited by selection bias in a non-randomized observational study. When compared to RT, cRP did not show a significant difference in OS and CSS, but it exhibited a better 2-year local event-free survival (LEFS) at 92% compared to 77% for RT-treated patients [42].

Cytoreductive radical prostatectomy was also significantly associated with longer castration-resistant-free survival at 3 years compared to the ADT alone group (59% vs. 40%, *p* = 0.02) [43]. The 3-year castration-free survival was very similar to the prospective ProMPT trial where patients with de novo oligometastatic prostate cancer who underwent cytoreductive prostatectomy had 3 yr OS of 87.9% and 3-yr CRPC-free survival of 65.6%. The study also showed the potential of pre- and post-operative circulating tumour cell numbers as a biomarker for improved treatment selection, with patients having two or more CTCs having worse OS and CRPC-free survival [44]. The TRoMbone trial was a randomized controlled trial that similarly looked at the impact of cytoreductive radical prostatectomy in a cohort of oligometastatic prostate cancer patients. While cRP seemed to be a viable and safe treatment option with minimal impact on quality of life, the available data primarily focused on early oncological outcomes, specifically positive surgical margins and postoperative PSA levels. The study reported a PSM rate of 42%, and a notable 83% of patients achieved a postoperative PSA value of less than 1 ng/mL within six months [45].

In the local treatment of oligometastatic prostate cancer patients, it is also important to consider the attendant morbidity especially with cytoreductive surgery given its invasive nature. In the FUSCC-OMPCa trial, the overall 90-day post-operative complication rate was 28%, of which 3.5% had Clavien-Dindo grade > 3a. The 2-year incontinence rate was 5% and <13% of patients who experienced long-term, grade 3–4 side effects such as urgency and hot flushes. Lumen et al. reviewed the rate of invasive treatment of the urinary tract (including catheterization) in de novo low-volume mHSPC patients from the LoMP registry as an arbiter of complications from local disease progression or cytoreductive prostatectomy or radiation therapy of the primary tumour. Patients who underwent cytoreductive radical prostatectomy had a significantly lower risk of invasive treatment needed compared to those who underwent radiation therapy to the primary tumour (HR 0.31, 95% CI 0.11–0.86; *p* = 0.024) and when compared to those who only had systemic treatment (HR 0.25, 95% CI 0.10–0.64; *p* = 0.004). A significantly higher proportion of patients avoided invasive treatments at 2 years. Notwithstanding the lack of a randomized controlled design, the results from the LoMP registry suggested that surgical cytoreduction offers the best approach for reducing local complications, however, at the expense of stress urinary incontinence. Close to 80% of patients were continent 1 year after surgery and only two (4.2%) patients required an artificial urinary sphincter. There were no Clavien-Dindo grade 4 or 5 complications observed [42,43]. The TRoMbone trial reported functional outcomes and safety at 6 months post cytoreductive surgery. Four patients (16.8%) had peri-operative complications and the overall continence rate at 6 months was 83.3%. There were no differences in the QoL scores between the SOC and cytoreductive radical prostatectomy. Erectile dysfunction, the other functional impact conventionally associated with radical prostatectomy, would be less relevant in a cohort of patients where systemic treatment and castration are the standard of care.

There are encouraging oncological outcomes for surgical removal of the prostate primary in low-volume mHSPC, although more high-quality data are awaited. The functional outcomes, in particular continence rates, are comparable in these series to those seen in the primary treatment of localized prostate cancer. Importantly, an understated utility and benefit of cytoreductive prostatectomy is in the local control of the disease. Locoregional symptoms are significant and can often be overlooked in the management of metastatic prostate cancer patients. Studies have shown that as much as two thirds of de novo mHSPC patients present with locoregional symptoms [46] and up to a third of patients with metastatic prostate cancer patients may require local palliative treatments [47]. Cyto-reductive prostatectomy in this setting may have added utility in the relief of urinary obstruction and haematuria, which can occur in up to 42.3% and 27% of patients with metastatic disease, respectively [47].

Despite the lack of randomized trials, current evidence has alluded to the usefulness of the local treatment of the primary tumour in advanced prostate cancer. Drawing lessons from local therapy in clinically node positive (cN1) prostate cancer, which has shown that both radical prostatectomy and radiation therapy leads to a significant improvement in survival outcomes compared to just observation or ADT alone, we can postulate that local therapy similarly may offer benefits in a select group of patients with a low metastatic burden [48].

### 4.5. Pelvic Lymph Node Dissection

As we prepare for the onslaught of patients with oligometastatic disease detected by next generation imaging, a significant area of prostate cancer management is being challenged. Extended pelvic lymph node dissection (ePLND) remains controversial on many fronts. The EAU guidelines recommend an ePLND for patients with a nomogram-predicted risk of lymph node invasion of >7%. However, such a strategy would subject the majority of patients to an unnecessary procedure with its attendant morbidity. More importantly, the oncological benefit of ePLND is uncertain.

With oligometastatic nodal disease detected in the pelvis, would this provide an opportunity for eradication of disease? There is evidence to show that even with an extended template, affected lymph nodes may be missed [49]. On the one hand, the high sensitivity of PSMA PET may allow us to spare patients unnecessary ePLND [50]. On the other hand, the finding of limited nodal metastases offers an opportunity to remove these diseased nodes during radical prostatectomy. The ability to localize these nodes with PSMA ligands labelled with gamma-emitting radionuclides such as 99m-technetium (e.g., 99mTc-PSMA-I&S) has moved the needle in terms of radio-guided surgery (RGS) for prostate cancer.

Radio-guided surgery (RGS), in a broad sense, is the use of radiation detection probes (i.e., handheld gamma probes) during surgery to identify radioactively labelled lesions inside the body with the aim of improving surgical outcome. The main drawback of PSMA PET lies in the inability to detect metastatic lymph nodes smaller than 4–5 mm, which relates to a technical limitation of the PET scanner. The use of an intra-operative gamma probe during radical prostatectomy offers the potential to improve the detection of small metastatic lymph nodes and potentially enhance surgical outcomes and clinical results with radio-guided surgery. RGS can be carried out either with single photon gamma emitting isotopes (i.e., Technetium-99 m or Indium-111) labelled with PSMA inhibitors using intra-operative gamma-ray probes or with beta-ray probes, detecting beta radiation emitted by routinely used PET radiopharmaceuticals (i.e., Gallium-68 or Fluorine-18) labelled with PSMA inhibitors. Early studies have shown that PSMA-RGS can be valuable in identifying lymph node involvement (LNI) in patients with a positive PSMA PET/CT scan undergoing salvage lymph node dissection [51,52]. In a recent interim analysis of a phase 2 prospective clinical trial involving intermediate to high-risk prostate cancer patients undergoing radical prostatectomy and RGS using 99mTc-PSMA I&S, the reported specificity on a per region and per patient basis was 99% and 100%, respectively [53]. However, the lack of sensitivity was also highlighted in this study (63% per region analysis; 67% per patient analysis). Importantly, this technique missed a case of micrometastatic LNI that was not detected by preoperative imaging in one patient, and the authors conclude that negative findings at PSMA-RGS should guide the surgeon towards more extended dissection in men with positive spots at the preoperative PSMA PET, to identify nodal metastases even outside the standard template [53].

Overall, this augmented strategy of pelvic lymph node dissection may bolster confidence in the oncological clearance of PSMA-detected nodal oligometastases in the pelvis. However, this technique is far from optimized as the size of the diseased lymph nodes continues to impact RGS, and the decreased uptake of radiopharmaceuticals particularly in micrometastases results in an insufficient signal-to-background ratio, making their detection difficult. In addition, the ability to resect positive lymph nodes in atypical locations is another purported utility of RGS and the probe design will need to allow for versatility and ease of manoeuvre during surgery. Lastly, the isotope used will need to be refined to allow a better signal-to-background ratio. Better precision can potentially be achieved with positron emitter detectors and readily available radiopharmaceuticals such as 68 Ga-PSMA-11, which offers a higher tumour-to-background ratio due to the shorter penetration depth of beta rays compared to gamma rays. The results from an ongoing phase 2 trial (NCT05596851) will further inform us of the feasibility and diagnostic performance of β + emission PSMA-RGS. Until then, further prospective studies are needed to consolidate the evidence and experience before its utility becomes more certain.

## 5. Conclusions

As we cautiously tread the landscape of OMPC currently, we are forced to have ambiguous interpretations of OMPC in the era of next generation imaging. Much like the hypothetical middle child syndrome, the entity of OMPC does not have established traits and receives much less attention. However, there remains a pressing need to define the optimal approach for this group of patients with a small metastatic burden with the intent to improve survival and even delay toxicities of systemic treatment. The current body of evidence seems to suggest three or less non-visceral metastatic lesions as the criteria of the ideal oligometastatic patient who would have PFS benefits with MDT, with or without systemic treatment, although the ideal modality for MDT remains uncertain. There is also evidence of a survival benefit for the local control of the primary tumour with radiotherapy in this similar patient population.

Our understanding of this disease state will undoubtedly deepen as data emerge from more prospective trials utilizing different modalities to capture OMPC and as we consolidate our understanding and acknowledge the concept of a low metastatic disease entity, one that will hopefully offer viable opportunities to reign in the disease before it escalates into a truly advanced disease.

## Figures and Tables

**Table 1:** Trials examining effect of MDT in OMPC [25,26].

Trial	Imaging Modality	Metachronous vs. Synchronous	Definition of Oligometastases	Metastases-Directed Therapy	Outcome
Conventional imaging					
ORIOLE [27]	CT, Bone Scan	Metachronous	≤3 bone or lymph node metastases	SABR vs. observation	Improved PFS at 6 months (19% vs. 61%, *p* = 0.005) Improved median PFS (not reached vs. 5.8 months; HR, 0.30; 95% CI, 0.11–0.81; *p* = 0.002)
RAVENS [26]	CT, Bone scan	Synchronous	≤3 (at least 1 bone metastasis)	SABR + radium-223 dichloride vs. SABR alone	Ongoing
PET/CT Scan [18,20,21,22,23]					
POPSTAR [24]	18F-NaF	Synchronous	≤3 bone metastases	SABR	1- and 2-year local-PFS was 97% (95% CI 91–100) and 93% (95% CI 84–100) Distant PFS was 58% (95% CI: 43–77) and 39% (95% CI: 25–60) 48% 2-year freedom from ADT
POPSTAR II [28]	68Ga-PSMA or 18F-DCFPyL PET/CT	Metachronous	1–5 sites of nodal or bony metastases	(SABR) alone or SABR plus 2 cycles of 177Lu-PSMA	Ongoing
STOMP [29]	Choline PET/CT	Metachronous	≤3 bone or lymph node metastases	Surveillance or MDT of all detected lesions (surgery or stereotactic body radiotherapy)	Median ADT-free survival was 13 months (80% CI, 12–17 months) for the surveillance group and 21 months (80% CI, 14–29 months) for the MDT group (HR 0.60 [80% CI, 0.40 to 0.90]; log-rank *p* = 0.11)
TROD 09-004 Study [30]	68Ga-PSMA PET/CT	36.5% had synchronous, and 47 (63.5%)	≤5 metastases	SBRT	2-year PCSS and PFS rates were 92.0% and 72.0%, respectively. PSA decline os 75.7% 64.9% had a PSA response (defined as at least 25% decrease in PSA after MDT) 2-year local control rate per lesion of 95.4%.
OLI-P [31]	Ga-68 PSMA-PET-CT	Metachronous	five or fewer lymph node or osseous metastases	SBRT	Median time to PSA progression of 13.2 months Median time to ADT of 20.6 months PSA progression-free rate of 21.4% after 3 yr
BULLSEYE [32]	18F-PSMA-PET-CT	Synchronous	≤5 metastases	^177^Lu-PSMA-617 (^177^Lu-PSMA-I&T)	Ongoing

## Data Availability

Data is contained within the article.
